# Comment on Siracusa et al. Fibromyalgia: Pathogenesis, Mechanisms, Diagnosis and Treatment Options Update. *Int. J. Mol. Sci.* 2021, *22*, 3891

**DOI:** 10.3390/ijms22169075

**Published:** 2021-08-23

**Authors:** Iván Cuyul-Vásquez, Felipe Araya-Quintanilla, Héctor Gutiérrez-Espinoza

**Affiliations:** 1 Departamento de Procesos Terapéuticos, Facultad de Ciencias de la Salud, Universidad Católica de Temuco, Temuco 4813302, Chile; Ivancuyul@gmail.com; 2 Rehabilitation in Health Research Center (CIRES), Universidad de las Americas, Santiago 7500975, Chile; 3 Faculty of Rehabilitation Sciences, Universidad Andres Bello, Santiago 7591538, Chile; kinehector@gmail.com

We have read the study by Siracusa et al. [1] with interest. We appreciate the effort of the authors to perform a literature review. However, we have detected some important departures and inconsistencies to which we would like to draw attention. They need to be discussed to avoid errors in future research or clinical practice.

First, regarding the pain conceptualization used in this review, the authors use unclear and unusual concepts, such as “pain sensitivity”, “transmission of pain signals”, “transmit pain”, and “pain signals”. All of these concepts are based on the understanding of pain as a synonym of nociception [2]. This is a viewpoint that considers pain as an input. However, the evidence describes pain as an output, the result of a complex evaluative process of environmental and multisystem information [2,3]. For this reason, currently, pain is considered a personal somatic experience in response to a threat to bodily or existential integrity [4]. A few years ago, researchers showed that pain and nociception are not synonymous concepts [3]. The perception of pain requires the ability to assess information from the environment, body receptors, past experiences, and memories of pain; as such, nociception is not the only pathway to generate pain [2,3]. For this, the International Association for the Study of Pain (IASP) has proposed the concept of nociplastic pain in subjects who have no clear evidence of actual or potential tissue damage [5]. Therefore, the authors must use an appropriate conceptualization and choose a good treatment in clinical practice.

Regarding the clinical implications of pain conceptualization, the authors mention “The notion that peripheral factors may underlie pain in fibromyalgia (FM) is supported by the observation that the administration of lidocaine into the muscles of patients with FM significantly reduced hyperalgesia at the local site, and moreover, the pain perceived outside the injection site was reduced by 38%”. This sentence is inappropriate, considering that the decrease in pain and hyperalgesia is mainly due to the reduction in “peripheral factors” through the specific effect of lidocaine. The study cited by the authors describes differences in favour of the lidocaine group on mechanical and thermal hyperalgesia, but no differences were observed between the lidocaine and saline groups for pain intensity [6]. The same results regarding pain intensity have been reported in other clinical trials [7,8]. However, it should be noted that the administration of injectable drugs has a considerable contextual effect, which has been estimated at 60% of the pain relief achieved with treatment in people with FM [9]. The discrepancies between clinical pain and hyperalgesia with lidocaine treatment have been scarcely reported in the literature, so further investigation is needed.

Second, regarding to the diagnosis, the authors mention “According to the American College of Rheumatology (ACR), the diagnosis of FM includes two variables: (1) bilateral pain above and below the waist, characterized by centralized pain, and (2) chronic generalized pain that lasts for at least three months, characterized by pain on palpation in at least 11 of 18 specific body sites”. The authors’ description refers to the ACR 1990 criteria that currently are not used because they have been observed to underdiagnose FM, are unstable over time, and focus the attention of the physician to allodynia and hyperalgesia only [10,11]. The ACR 1990 criteria fail to discriminate patients with FM from others with chronic widespread pain [11,12]. Thus, the ACR 1990 criteria are more of a severity assessment tool than a diagnostic tool [11,12]. For these reasons, it is not currently recommended to use the ACR 1990 criteria for a diagnosis of FM [13]. However, the combination of the ACR 1990 and 2010 criteria has allowed the identification of subgroups in patients with FM, but its clinical utility remains unclear, and further research is needed [14]. The current recommendation for the diagnosis of FM is based on the ACR 2016 criteria [15]. According to these, the diagnosis of FM is based on the presence of generalized pain as well as the scores of the Widespread Pain Index (WPI) and the Symptom Severity Scale (SSS) [15]. Thus, it is important that the authors rectify the information regarding the diagnosis of FM and explain the psychometric properties, strengths, and limitations of the assessment instruments.

Regarding inflammatory cytokines, the study mentions “The IL-8 expression pattern could aid in the diagnosis of fibromyalgia and in effective treatment strategies if confirmed in further studies”. However, the evidence is inconclusive on this topic [16,17,18]. Essentially, it is not feasible to make an association between pro-inflammatory cytokines and FM because when analysing the subgroups of patients, there are different covariates such as medication, gender, time of the blood sample, diet, and level of physical activity, which explain the heterogeneity and differences in these results. Therefore, this sentence is imprecise. Additionally, another study showed that the relationship between chronic widespread pain and cortisol levels in patients with FM is unclear [19].

Finally, we consider it relevant that the authors explicitly report the limitations of their research. In the review, we observed a selection bias in the studies in relation to the diagnosis, mechanism, and psychological factors of FM. Non-pharmacological interventions such as exercise, physical activity, education, and multimodal or multicomponent treatment were omitted in this review despite the existing recommendations and scientific evidence for these treatments in this group of patients [20,21,22,23,24,25,26].

## Data Availability

Not applicable.
